# Limited geographic distribution of the novel cyclovirus CyCV-VN

**DOI:** 10.1038/srep03967

**Published:** 2014-02-05

**Authors:** Le Van Tan, Menno D. de Jong, Nguyen Van Kinh, Nguyen Vu Trung, Walter Taylor, Heiman F. L. Wertheim, Arie van der Ende, Lia van der Hoek, Marta Canuti, Martin Crusat, Soeng Sona, Nguyen Hanh Uyen, Abhishek Giri, Nguyen Thi Thuy Chinh BKrong, Ho Dang Trung Nghia, Jeremy Farrar, Juliet E. Bryant, Tran Tinh Hien, Nguyen Van Vinh Chau, H. Rogier van Doorn

**Affiliations:** 1 Oxford University Clinical Research Unit, Ho Chi Minh City, Vietnam; 2 Department of Medical Microbiology, Academic Medical Center, University of Amsterdam, Amsterdam, the Netherlands; 3 National Hospital for Tropical Diseases, Hanoi, Vietnam; 4 Hanoi Medical University, Hanoi, Vietnam; 5 Oxford University Clinical Research Unit, Hanoi, Vietnam; 6 The Netherlands National Reference Laboratory for Bacterial Meningitis, Amsterdam, the Netherlands; 7 Centre for Tropical Medicine, Nuffield Department of Medicine, University of Oxford, Oxford, UK; 8 Angkor Hospital for Children, Siem Reap, Kingdom of Cambodia; 9 Oxford University Clinical Research Unit, Patan Hospital, Kathmandu, Nepal; 10 Hospital for Tropical Diseases, Ho Chi Minh City, Vietnam; 11 Pham Ngoc Thach University of Medicine, Ho Chi Minh City, Vietnam

## Abstract

A novel cyclovirus, CyCV-VN, was recently identified in cerebrospinal fluid (CSF) from patients with central nervous system (CNS) infections in central and southern Vietnam. To explore the geographic distribution of this novel virus, more than 600 CSF specimens from patients with suspected CNS infections in northern Vietnam, Cambodia, Nepal and The Netherlands were screened for the presence of CyCV-VN but all were negative. Sequence comparison and phylogenetic analysis between CyCV-VN and another novel cyclovirus recently identified in CSF from Malawian patients indicated that these represent distinct cycloviral species, albeit phylogenetically closely related. The data suggest that CyCV-VN has a limited geographic distribution within southern and central Vietnam. Further research is needed to determine the global distribution and diversity of cycloviruses and importantly their possible association with human disease.

Cycloviruses (CyCVs) belong to the *Circoviridae* family and have recently been found in different sample types from different hosts, including mammals and insects^1^,^2^,^3^,^4^,^5^. Recently, we reported a new cyclovirus species, tentatively named cyclovirus-Vietnam (CyCV-VN), in cerebrospinal fluid (CSF) of two Vietnamese patients^6^. The virus was subsequently detected in 4% of 642 CSF samples of patients with central nervous system (CNS) infections from 7 different provinces in southern and central Vietnam, but in none of 122 CSF samples from patients with noninfectious CNS conditions^6^. Almost simultaneously, another novel CyCV (CyCV-VS5700009) was reported in CSF and serum from patients with paraplegia in Malawi^7^, while other CyCVs have previously been reported in stool samples of patients with acute flaccid paralysis from Tunisia, Pakistan and Nigeria^3^. Together, these data suggest that cycloviruses may have a wide geographic distribution, and that specific CyCV species might be associated with specific clinical phenotypes, although it should be noted that associated pathology of cycloviruses has yet to be proven.

## Results

To explore whether CyCV-VN is circulating beyond central and southern Vietnam, we screened a total of 615 CSF specimens from patients with acute CNS infections from northern Vietnam (n = 233), Cambodia (n = 123), Nepal (n = 80) and The Netherlands (n = 179) for the presence of CyCV-VN DNA (Table 1).

CyCV-VN DNA, however, was absent from all 615 CSF specimens tested, suggesting a confined geographical distribution of this virus.

**Figure 1:**
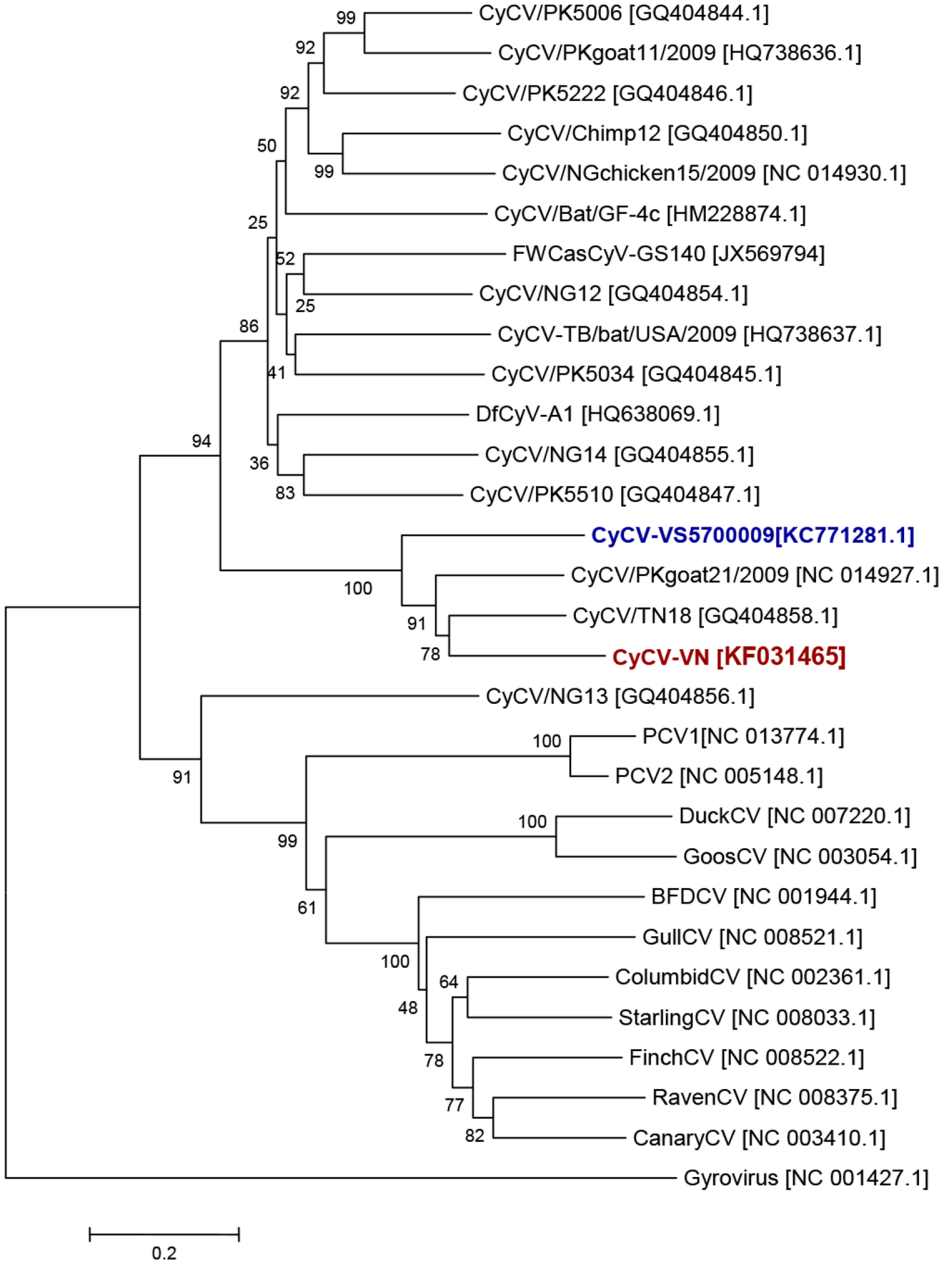
Reconstructed phylogeny tree of complete genome sequences of cycloviruses, CyCV-VN (red) and CyCV-VS5700009 (blue); circoviruses and a gyrovirus were used as outliers. CV: circovirus, Sequence accession number are in square brackets.

To determine whether CyCV-VN represents a cycloviral species distinct from CyCV-VS5700009, the novel cyclovirus recently detected in CSF from Malawian patients^7^, we conducted sequence comparisons and phylogenetic analyses to examine the genetic relationship between CyCV-VN and CyCV-VS5700009. Pairwise comparisons showed that the degree of sequence similarity between CyCV-VN and CyCV-VS5700009 was 60% at the nucleotide level of the complete genome sequence and 36% and 76% at the amino acid level of the capsid protein and replication association proteins, respectively. These values are all well below the proposed demarcation criteria to distinguish different CyCV species (75%, 70% and 85% identity, respectively)^3^. The genetic distance between the two viruses was confirmed by phylogenetic analysis based on complete genome sequences (Figure 1). These analyses indicate that CyCV-VN and CyCV-VS5700009 represent two distinct cycloviral species.

## Discussion

In a previous study we showed that the newly discovered CyCV-VN was widely distributed in central and southern Vietnamese provinces^6^. In this study we further explored the geographic distribution of CyCV-VN by testing 615 CSF samples from patients with suspected CNS infections from northern Vietnam, Cambodia, Nepal and the Netherlands. CyCV-VN was absent from all 615 tested CSF samples from these locations, suggesting that the geographic distribution of CyCV-VN may be limited to southern and central Vietnam.

**Figure 2:**
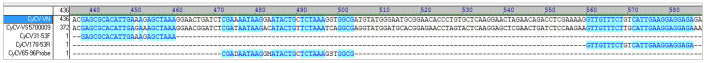
Nucleotide sequence alignment showing sequence identity between primers/probe of CyCV-VN PCR used and CyCV-VN- and CyCV-VS5700009 sequences; degenerate nucleotides: D: A, G or T; M: A or C; S: G or C.

We previously reported a prevalence of 4% (28/644) among children and adults with confirmed or suspected CNS infections in overlapping time periods with the current study^6^, hence failure to detect CyCV-VN in this study is unlikely due to sample size restrictions or temporal bias. This is supported by observed statistically significant differences in prevalence (Table 1). However, it cannot be ruled out that related cycloviruses (including CyCV-VS5700009) may have gone undetected since our PCR method was designed for specific detection of CyCV-VN^6^. Indeed, sequence analysis indicates that the differences at primer and probe binding sites would render detection of the Malawian virus and other reported cycloviruses by CyCV-VN-specific PCR method highly unlikely (Figure 2)^6^. Testing for other (un)known cycloviruses in these 615 CSF samples is, therefore, desirable, but beyond the scope of the present study. Likewise, to fully explore the circulation of CyCV-VN in the regions from where 615 patients came it is necessary to test for the CyCV-VN in livestock species (e.g. pigs, chickens and ducks) that have been previously tested positive for the virus in more than 50%^6^.

In summary, our new data from clinical case detections suggest that circulation of CyCV-VN is restricted to southern and central Vietnam. Further research utilizing degenerate PCR primers is needed to examine to what extent diverse cycloviruses can be detected in CSF of patients with CNS infections worldwide. In addition, further investigations of cyclovirus prevalence in healthy subjects, clinical cases, and non-human species will be required to assess the epidemiological significance of these findings.

## Methods

### Clinical materials

CSF specimens analyzed in this study were derived from three prospective clinical studies designed to establish the etiology of CNS infections in northern Vietnam (n = 233), Cambodia (n = 123) and Nepal (n = 80), The Dutch CSF specimens (n = 179) represented a random sample of anonymized specimens from Dutch patients with suspected CNS infections submitted to the Netherlands Reference Laboratory for Bacterial Meningitis in Amsterdam.

### Nucleic acid extraction

As part of standard operating procedures applied in our laboratory, CSF samples were spiked with an optimal amount of phocid herpesvirus and equine arteritis virus as (reverse transcription) PCR internal controls^8^,^9^, and were then subjected to an automatic extraction procedure with use of the Viral NA Small Volume Kit (Roche applied sciences, UK) and a MagNA Pure 96 system (Roche applied sciences). The isolated nucleic acid were then stored at −80°C for subsequent PCR screening for CNS pathogens as well as CyCV-VN.

### Real time PCR

PCR screening of CyCV-VN was performed on extracted nucleic acid samples that had been tested by PCRs for CNS pathogens and had expected crossing point values of internal controls between 33 and 35, and was carried out as previously described^6^. In brief, the PCR was done in a final 25 μl volume reaction containing 1.25 U of HotstarTaq DNA polymerase (QIAgen GmbH, Hilden, Germany) 800 μM of each primer, 100 μM of a FAM-labeled probe, 400 μM each dTNPs 5 mM of MgCl_2_ (provided with the polymerase), 1 × PCR buffer (provided with the polymerase), and 3 μl of extracted NAs. PCR reaction was performed on a LightCycler 480 Instrument II (Roche applied Sciences, UK) with an initial polymerase-activation step at 95°C for 14 min 30 sec followed by 45 cycles of 95°C for 30 sec, 55°C for 30 sec and 72°C for 30 sec. A positive control and several negative controls (including extraction controls and water controls) were included in every PCR run. All the PCR experiments were performed in molecular diagnostic facilities that consist of three physically separated laboratories for reagent preparation, extraction and amplification, and these were used a unidirectional workflow.

### Sequence analysis

Pairwise comparisons and phylogenetic analyses were done using AlignX (Vector NTI Advance 7; Invitrogen, Carlsbad (CA), USA) and neighbor-joining in MEGA version 4^10^.

### Ethical statement

All clinical studies from which the CSF samples were chosen for PCR screening in the present study were reviewed and approved by the Institutional Review Boards of collaborating hospitals and the Oxford Tropical Research Ethics Committee (OxTREC), University of Oxford, United Kingdom. Informed consent was obtained from patients or a parent or guardian of each enrolled patients.

## Author Contributions

H.D.T.N., N.V.V.C., N.V.K., W.T., H.F.L.W., A.v.d.E., A.G., S.S. and T.T.H. collected CSF specimens. M.C., N.V.T., N.H.U., M.C., J.B. and N.T.T.C.B. conducted laboratory experiments. L.V.T. did sequence analyses. L.V.T., H.R.v.D., J.F., L.v.d.H., J.B. and M.D.d.J. designed the study. L.V.T., H.R.v.D. and M.D.d.J. drafted the manuscript. All authors were involved in subsequent reviewing and editing the manuscript.

## Figures and Tables

**Table 1:** Patient cohorts from which samples were used in this study to detect CyCV-VN

	Number of samples		Population	City*	Country	References	Time frame	*P* value#
	total	positive						
**Previous study**	642	26			southern and central Vietnam	6	1999–2009	
**Present study**	233	0	adults	Ha Noi	northern Vietnam	11	2007–2008	0.0004
	123	0	children	Siem Reap	Cambodia	12	2009–2010	0.01
	80	0	all ages	Kathmandu	Nepal	13	2009–2011	0.1
	179	0	all ages	nationwide	The Netherlands	unpublished	2010–2011	0.003
**All**	615	0						<0.0001

*Name of the city indicates the location of the hospital where the patients were admitted.

^#^Fischer exact; compared with results from the study in reference #6.
